# Editorial: The placenta, fetomaternal tolerance and beyond: A tribute to Sir Peter Medawar on the 60^th^ anniversary of his Nobel Prize

**DOI:** 10.3389/fimmu.2022.1021885

**Published:** 2022-09-20

**Authors:** Gabriela Barrientos, Maria Emilia Solano, Sandra M. Blois, Surendra Sharma

**Affiliations:** ^1^ Laboratorio de Medicina Experimental, Hospital Alemán. Consejo Nacional de Investigaciones Científicas y Técnicas (CONICET), Ciudad Autónoma de Buenos Aires, Argentina; ^2^ Laboratory for Translational Perinatology- Focus: Immunology, University Department of Obstetrics and Gynecology, University Hospital Regensburg, Regensburg, Germany; ^3^ Department of Obstetrics and Fetal Medicine, University Medical Center Hamburg-Eppendorf, Hamburg, Germany; ^4^ Department of Pediatrics, Women and Infants Hospital-Warren Alpert Medical School of Brown University, Providence, RI, United States

**Keywords:** placenta, Peter Medawar, pregnancy, fetomaternal tolerance, inflammation

## Sir Peter Medawar era and beyond

**Figure 1:**
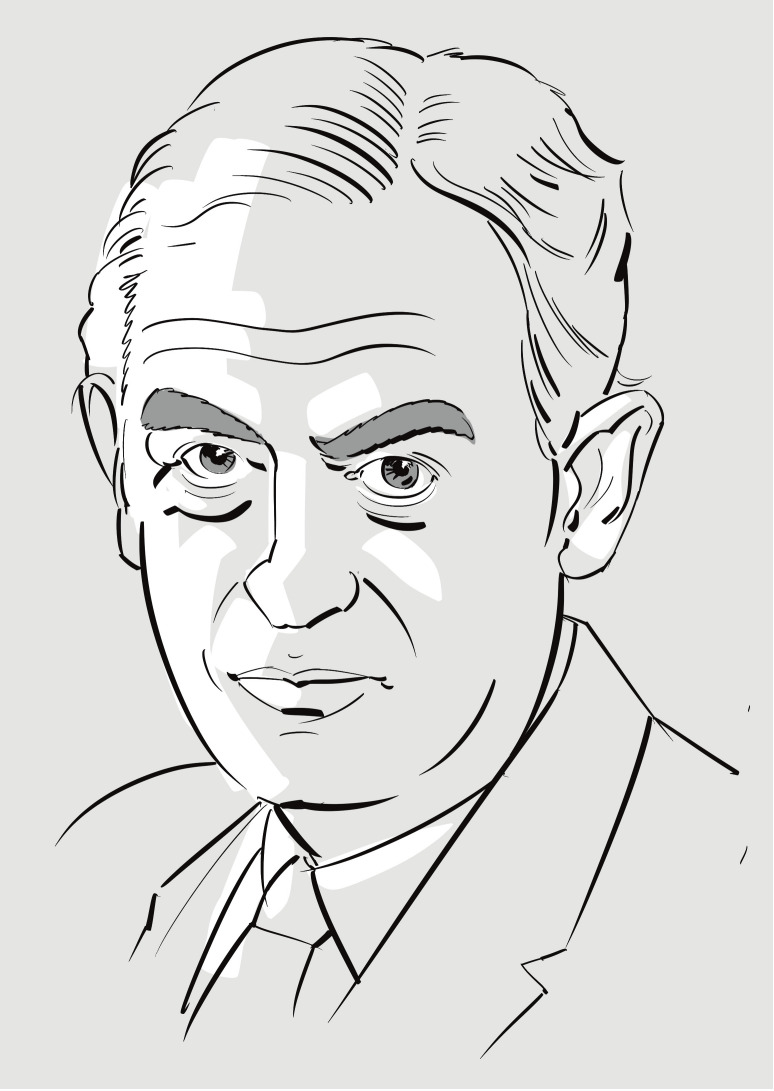
Sir Peter Medawar: 1915-1987 and Beyond.

Sir Peter Medawar revolutionized our knowledge about how a semi-allograft fetus can evade maternal immune system (**Figure 1**) (1). Although the old concepts of tissue transplantation as he considered them may not entirely apply to our current understanding of fetal immune tolerance, his Nobel Prize winning ideas provided us a platform to build on the new paradigms to understand the intricately choreographed orchestration of immunity at the maternal-fetal interface. The pregnant uterus is amply replete with immune cells which theoretically should reject the fetus. However, non-cytotoxic cross talk between specialized uterine immune cells and the placenta is thought to be a critical factor in maintaining local immune tolerance. On the other hand, among the vulnerable populations, pregnant women and their fetuses have traditionally represented a high-risk population during viral pandemics and in response to stress factors and intrauterine microbial infections in general. These events are likely to induce altered maternal immunity and effect on gametogenesis and organogenesis as well as placental functions. Adverse pregnancy outcomes are on the rise globally and to date, no clear mechanistic underpinnings or therapeutic options are available. Importantly, long-term effects of *in utero* exposure to viral or bacterial products and of severe pregnancy complications are still not known. A question that also remains unaddressed is whether *ex-vivo* recapitulation is possible to understand the pregnancy continuum involving inflammation-associated implantation through the anti-inflammatory phase of pregnancy and inflammation-associated parturition.

This Research Topic on the placenta and fetomaternal tolerance during pregnancy centers around consequences for Normal Pregnancy and Maternal and Neonatal Health and presents cutting edge information on various aspects of basic systemic and uterine immunity, placenta as a regulatory and infectious target, and clinical observations in pregnant women. The articles included in this collection provide knowledge that assimilates the current understanding and goes beyond the Medawar era. We hope this issue will be of benefit to researchers globally, interested in the role of cutting-edge concepts that have been put forward to unravel mechanisms that underlie pregnancy complications.

## Urgent need for contemporary and comprehensive information on maternal tolerance in pregnant women

Over the years, there has been an explosion of publications on fetomaternal immune tolerance in general, including those reporting on clinical consequences for pregnant women. However, the information to the general public has suffered from fragmented reports and data derived from mice and humans sometimes not corroborating. Clinical evaluation of the mechanisms derived from animal models has been very helpful but still needs validation from human models. In this context, state of the art technologies such as whole genome sequencing and single-cell proteomics provide invaluable tools to gain insight into the immunological landscape throughout the pregnancy continuum, from the embryo-endometrial crosstalk driving implantation and aberrant immune interactions in preeclampsia, to the signals promoting the onset of labor and the maturation of the immune system in preterm born infants as illustrated by original research featured in this collection. The twenty-three manuscripts comprised in this Research Topic cover most of the cutting-edge information on important issues relating to pregnancy, immune cells, placenta, cytokines, hormones, extracellular vesicles, and neonatal health. Each review or original article is authored by experts on the specific topic of their respective research and/or clinical work.

## Infections, immunology and consequences

The uniqueness of the uterine mucosal lining relies in that it provides a nurturing environment to the fetal allograft while at the same time remaining competent to mount an effective immune response towards infections. Articles within this Research Topic also addressed the mechanisms involved in the uterine response against viral infections, including observational studies reporting an inefficient placental infection by SARS-CoV-2 and poor manifestation of maternal and neonatal immune responses. These observations relate to the question whether pregnancy contributes to controlled or severe COVID-19 disease. Unlike other coronaviruses, SARS-CoV-2 infected pregnant women remain asymptomatic with rare incidence of mortality. Also, even in the post Medawar era, the question as to how maternal immune cells are educated to support pregnancy and the identity of the signals triggering immune rejection remain major challenges. Novel findings on antigen presenting cells, maternal HLA Ib polymorphisms, allorecognition by uterine NK cells and type 1 CD8+ T cells shed light into these important questions. On the other hand, new observations suggest that certain immune cells, for example mucosal-associated invariant T cells, and bacterial components, albeit with low abundance, may function as anti-microbial defense and support pregnancy development, respectively. This Research Topic also includes important contributions of cytokines, carbohydrate Lewis antigens, glycan binding proteins, adhesion molecules, HLA-DR antigens, and placental inflammasome in programming a continuum of pregnancy complications including preeclampsia, gestational diabetes mellitus, fetal growth restriction and preterm birth.

## Clinical consequences of aberrant uterine immunity on maternal and neonatal health

Several manuscripts have been devoted to review the current data and conceptualize the various factors integral to the understanding of diagnostic challenges, therapeutic controversies, intrauterine transmission, and maternal and neonatal complications. Among these, contributions on CD8+ T cells and decidual NK cells provide an updated perspective on immune cell biology at the maternal fetal interface and the impact on health and disease. Together with articles illustrating current knowledge on glycoimmune interactions, immunosuppressive signatures expressed by placentally derived extracellular vesicles and their synergy with microchimeric cells, we have curated a comprehensive outline of the intricate mechanisms governing fetomaternal tolerance covering the entire gestation period from disease severity, management considerations for care of severe and critically ill women, role of co-infections, and prenatal care and labor.

We hope that manuscripts included in this Research Topic will provide cutting edge insights for diverse aspects of placental microenvironment in pregnant women and its effects on maternal and neonatal health.

## Author contributions

SS conceptualized the work, SS and GB drafted the first version. SB designed the illustrations included in this work. All authors contributed to the final version and approved it for publication.

## Funding

This work was supported by grants PICT-2020-03263 (GB), Deutsche Forschungsgemeinschaft grants SO1413/3, SO1413/5 to MS, Deutsche Forschungsgemeinschaft research grants (BL1115/2-1, BL1115/4-1, BL1115/8-1; Heisenberg program BL1115/7-1. BL1115/11-1), and Heike Wolfgang Mühlbauer Stiftung to SB. NIH grants P20 GM121298 and 3P20GM121298-04W1 to SS.

## Acknowledgments

We would like to thank Prof. Elizabeth Simpson, the authors, and reviewers who contributed to the success of this Research Topic. Among them, the editors and many authors want to express their gratitude to Dr. Gerard Chaouat (1944-2021) who not only kept the flame of Sir Peter Medawar’s scientific legacy burning over the last decades. With his collegial generosity, influence on current and future generations, research, and ideas he shaped the progress of the field, and also of his Research Topic to which he contributed as reviewer for two manuscripts. We thank Björn von Schlippe and Fangqi Zhao for helping us with the illustrations.

## Conflict of interest

The authors declare that the research was conducted in the absence of any commercial or financial relationships that could be construed as a potential conflict of interest.

## Publisher’s note

All claims expressed in this article are solely those of the authors and do not necessarily represent those of their affiliated organizations, or those of the publisher, the editors and the reviewers. Any product that may be evaluated in this article, or claim that may be made by its manufacturer, is not guaranteed or endorsed by the publisher.
